# Using CRISPR/Cas9 to Knock out Amylase in Acinar Cells Decreases Pancreatitis-Induced Autophagy

**DOI:** 10.1155/2018/8719397

**Published:** 2018-05-17

**Authors:** Kohei Yasunaga, Tetsuhide Ito, Masami Miki, Keijiro Ueda, Takashi Fujiyama, Yuichi Tachibana, Nao Fujimori, Ken Kawabe, Yoshihiro Ogawa

**Affiliations:** ^1^Department of Medicine and Bioregulatory Science, Graduate School of Medical Sciences, Kyushu University, Fukuoka, Japan; ^2^Department of Molecular Endocrinology and Metabolism, Graduate School of Medical and Dental Sciences, Tokyo Medical and Dental University, Tokyo, Japan

## Abstract

Pancreatic cancer is a malignant neoplasm that originates from acinar cells. Acinar cells get reprogrammed to become duct cells, resulting in pancreatic cancer. Pancreatitis is an acinar cell inflammation, leading to “impaired autophagy flux”. Pancreatitis promotes acinar-to-ductal transdifferentiation. Expression of amylase gets eliminated during the progression of pancreatic cancer. Amylase is considered as an acinar cell marker; however, its function in cells is not known. Thus, we investigated whether amylase affects the acinar cell autophagy and whether it plays any role in development of pancreatitis. Here, we knocked out* ATG12* in a pancreatic cancer cells and acinar cells using CRISPR/Cas9. Autophagy inhibition led to an increase in the expression of duct cell markers and a simultaneous decrease in that of acinar cell markers. It also caused an increase in cell viability and changes in mitochondrial morphology. Next, we knocked out amylase in acinar cells. Amylase deficiency decreased autophagy induced by pancreatitis. Our results suggest that amylase controls pancreatitis-induced autophagy. We found that eliminating amylase expression contributes to pancreatic cancer etiology by decreasing autophagy. Furthermore, our results indicate that amylase plays a role in selective pancreatitis-induced autophagy of pancreatic enzyme vesicles.

## 1. Introduction

The pancreas is primarily composed of acinar cells, which are parenchymal cells, but is also made up of other cells such as duct cells. Acinar cells produce digestive enzymes, including pancreatic alpha amylase (AMY2) and trypsin. Pancreatic cancer (pancreatic ductal adenocarcinoma) is the most frequent type of malignant pancreatic neoplasm [1, 2]. Morphologically, pancreatic cancer exhibits distinctive features of duct cells. Thus, scientists previously hypothesized that pancreatic cancer originates from duct cells. However, pancreatic cancer was found to be an acinar cell-derived malignant neoplasm [3]. Acinar cells undergo reprogramming known as “acinar-to-ductal metaplasia” because of inflammation caused by pancreatitis and genetic mutations [3–8]. During this reprogramming process, acinar cells lose their acinar cell phenotype and acquire a duct cell phenotype. Reprogrammed acinar cells develop into pancreatic cancer. These processes are important in pancreatic cancer etiology, with pancreatitis contributing to the first step of this process.

Pancreatitis is a risk factor for pancreatic cancer in humans and is mainly characterized by acinar cell inflammation [1, 9]. Acinar cell inflammation is activated by trypsinogen inside of cells [10, 11]. Trypsinogen activation occurs via “impaired autophagy flux”, which causes a disease state similar to pancreatitis in mice [12–14]. Furthermore, autophagy deletion increases acinar-to-ductal metaplasia in mice [15, 16]. Autophagy leads to the development of pancreatitis and contributes to pancreatic cancer development via acinar-to-ductal metaplasia.

During autophagy, cells degrade and recycle long-lived proteins and dysfunctional mitochondria [17]. A reduction in autophagy leads to accumulation of dysfunctional mitochondria and alters cellular metabolism. In humans, an increase in autophagy corresponds to the poor prognosis of pancreatic cancer patients [18]. However, there is no evidence that targeting autophagy is effective for pancreatic cancer treatment. Previously, two major studies employed knockdown of autophagy in a human cell line and autophagy knockout in a genetically engineered mouse model (GEMM). Autophagy knockdown in the human cell line suppressed the progression of pancreatic cancer [19]. Thus, an autophagy-inhibiting agent as a drug for pancreatic cancer treatment was clinically tested [20]. However, autophagy knockout in the GEMM was associated with increased tumor-related death [16]. To reevaluate the relationship between autophagy and pancreatic cancer, we used the CRISPR/Cas9 system [21, 22] to knock out autophagy in a human pancreatic cancer cell line.

Next, we focused on loss of the acinar cell phenotype in the progression from pancreatitis to pancreatic cancer, specifically the loss of amylase expression. Pancreatic alpha amylase (AMY2) is an acinar cell marker and diagnostic marker for pancreatitis [23–25]. Pancreatic alpha amylase is a pancreatic enzyme produced only in acinar cells. The substrate of pancreatic alpha amylase is starch derived from plants, but its intracellular function is unknown. During reprogramming of acinar cells into duct cells in pancreatic cancer, pancreatic alpha amylase expression is lost. We hypothesized that the loss of pancreatic alpha amylase expression not only is a marker of loss of the acinar phenotype, but also plays a role in reprogramming these cells, particularly for autophagy.

## 2. Materials and Methods

### 2.1. Materials

The plasmid pSpCas9(BB)-2A-GFP (PX458) was obtained from Addgene (F. Zhang, #48138; Cambridge, MA, USA).

For immunoblotting, antibodies against amylase (sc-12821; Santa Cruz Biotechnology, Santa Cruz, CA, USA), LC3 (sc-271625; Santa Cruz Biotechnology), ATG12 (#2010; Cell Signaling Technology), and *β*-actin (013-24553; Wako Pure Chemical, Osaka, Japan) were used. Anti-mouse (sc-2031; Santa Cruz Biotechnology) and anti-goat (sc-2020; Santa Cruz Biotechnology) peroxidase-conjugated immunoglobulin were used as secondary antibodies. For immunofluorescence, an anti-LC3 monoclonal antibody (clone: #-1703, CTB-LC#-2-IC; Cosmo Bio, Tokyo, Japan), anti-tom20 (D8T4N; Cell Signaling Technology, Danvers, MA, USA), and Alexa 488-conjugated goat anti-mouse IgG (A11029; Thermo Fisher, Waltham, MA, USA) were used.

RIMI1640 (189-02025; Wako Pure Chemical) was used as a medium for AR42J cells and DMEM (044-29765; Wako Pure Chemical) was used as a medium for MIA PaCa-2 cells. Additionally, 10% fetal bovine serum (SH 30088.03; GE Life Sciences, Little Chalfont, UK) and 1% penicillin-streptomycin (15070063; Gibco, Grand Island, NY, USA) were added to the media. Phosphate-buffered saline (PBS) (10010-023; Gibco) was used for cell starvation.

### 2.2. Establishment of KO Cell Lines

CRISPR guide RNAs (gRNA) sequence targeting each gene were cloned into pX458 [26]. CRISPR Design (http://crispr.mit.edu/) was used to design the gRNA. A sequence with no high-scoring off-targets was selected. The selected target sequences were human ATG12- GGCTCCGGGGTGGTTGTTTC, rat ATG12- CCGGGAGGTTCCTCCGTAC, and rat amylase- AGTAATGTCAAGTTACCGATGGG. The primers used for cloning were human ATG12- 5′- CACCGGCTCCGGGGTGGTTGTTTC-3′ and 5′-AAAC GAAACAACCACCCCGGAGCC-3′, rat ATG12- 5′-CACCG CCGGGAGGTTCCTCCGTACC-3′ and 5′-AAACGGTACGGAGGAACCTCCCGGC-3′, and rat amylase- 5′-CACCGAGTAATGTCAAGTTACCGAT-3′ and 5′-AAACATCGGTAACTTGACATTACTC-3′.

MIA PaCa-2 and AR42J were transfected with pX458 with the above gRNAs inserted using Lipofectamine 2000 (Thermo Fisher). After 48 h, green fluorescent protein positive cells were isolated using a cell sorter (BD FACSARIA 3; BD Biosciences, Franklin Lakes, NJ, USA) and single clones were obtained. Clones with mutations in both alleles were identified by immunoblotting and confirmed by sequencing of genomic DNA.

### 2.3. Quantitative Reverse Transcription PCR

mRNA was extracted using Rneasy Mini Kit (Qiagen, Hilden, Germany). cDNA was produced using PrimeScript RT Master Mix (Takara, Shiga, Japan). The reaction conditions were 50 min at 50°C, followed by 5 min at 85°C. Quantitative analysis of expression was conducted using SYBR Premix Ex Taq (Takara, Shiga, Japan). The reaction conditions were 40 cycles for 15 s at 95°C and 1 min at 60°C. Expression was quantified by the ΔΔCT method using a 7500 fast (Applied Biosystems, Foster City, CA, USA). The primers used were human gapdh- ACATGTTCCAATATGATTCCA and TGGACTCCACGACGTACTCAG, human CK19- GCAGGTCCGAGGTTACTGAC and CCAGTGTGTCTTCCAAGGCA, rat gapdh- GTTACCAGGGCTGCCTTCTC and GGGTTTCCCGTTGATGACC, rat PTF1- CAGGTAACCAGGCCCAGAAG and TTTCATCAGCCCAGGAAAGG, and rat amylase- GCAACCAAGTGGCTTTTAGC and CAGTATGTGCCAGCAGGAAG.

### 2.4. Immunofluorescence Staining

Cells were fixed in 4% paraformaldehyde, washed three times with phosphate-buffered saline (PBS) and 50 *μ*g/mL digitonin/PBS for 5 min at room temperature, and blocked with 3% bovine serum albumin/PBS. Cells were then incubated for 1 h with a primary antibody at room temperature, followed by incubation for 1 h with a secondary antibody conjugated to green fluorescent protein at room temperature. Images were acquired using a Zeiss LSM 700 confocal microscope (Carl Zeiss, Oberkochen, Germany) using the ×63 objective. The pinhole size was 1 AU.

### 2.5. Cell Viability Assay

Cells were incubated with 10% alamarBlue for 1 h at 37°C in the presence of 5% CO_2_. After each stimulation, the same well was incubated again in medium containing 10% alamarBlue for 1 hr. Fluorescence was measured at an excitation/emission wavelength of 570/590 nm. “Δ fluorescent intensity” is defined as basal fluorescent intensity minus fluorescent intensity after stimulation. “Fluorescent intensity change (%)” is defined as fluorescent intensity after stimulation divided basal fluorescent intensity.

### 2.6. Statistical Analysis

An unpaired two-tailed *t*-test was conducted. Variance was confirmed to be roughly equal in both groups by an *F* test. Statistical processing was conducted using GraphPad Prism 7 software (GraphPad, Inc., Chicago, IL, USA).

## 3. Results

### 3.1. Autophagy Deletion Pancreatic Cancer Cells

**Figure 1:**
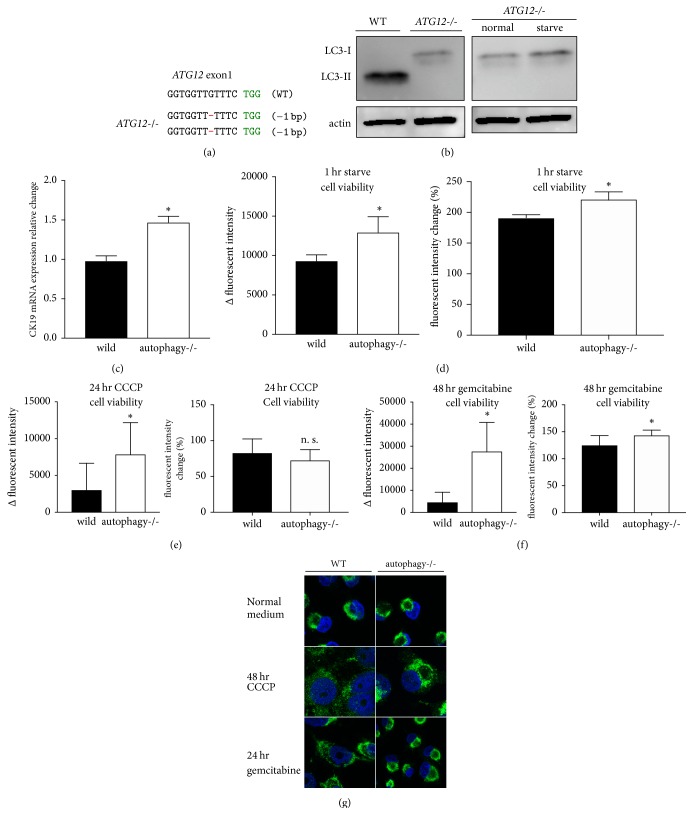
Autophagy deletion pancreatic cancer cells. (a)* ATG12*-deficient MIA PaCa-2 genomic DNA sequence. Bases shown in red are indels (insertions and deletions). PAM sequence is shown in green. (b) Lysate of wild-type and* ATG12*-deficient MIA PaCa-2 cells. Starvation was induced by culturing the cells in PBS for 1 hr. LC3-II is a marker of autophagy. Actin was used as a loading control. (c) CK19* (KRT19)* mRNA quantitative reverse transcription PCR. The vertical axis is the fold change relative to the GAPDH control. (d) Change in alamarBlue fluorescence after 1 h of starving. (e) alamarBlue fluorescence at 24 h with 10 *μ*M of CCCP added to the culture. (f) Change in fluorescence at 48 h, when 500 nM gemcitabine was added. (g) Confocal microscopy of immunofluorescent staining. Mitochondria are in green; Hoechst stain is in blue. ^*∗*^a value of *P* < 0.05 represents statistical significance.

To clarify the role of autophagy in pancreatic cancer, we established an autophagy deletion MIA PaCa-2 cell line. Autophagy consists of a conjugation system that requires LC3 (ATG8) and ATG12 [27, 28]. We edited exon 1 of* ATG12* in MIA PaCa-2 cells using CRISPR/Cas9. The edited genome sequence is shown in Figure 1(a). The western blotting results for the ATG5-ATG12 complex are shown in Supplemental Figure 1A.* ATG12*-deficient MIA PaCa-2 cells were subjected to LC3 western blotting and immunofluorescence staining to confirm autophagy deficiency. MIA PaCa-2 cells have increased LC3-II, an autophagy marker under normal conditions [19].* ATG12*-deficient cells exhibited no conversion from LC3-I to LC3-II (Figure 1(b)). In addition, during starvation, a state that generally induces autophagy, no conversion to LC3-II was observed (Figure 1(b)). LC3 was stained diffusely and LC3 puncta decreased in* ATG12*-deficient cells (Supp.  Figure 1B). Next, to evaluate the influence of autophagy deletion on inducing the duct phenotype, we measured the mRNA expression of the duct cell marker CK19* (KRT19)*. CK19 mRNA expression in autophagy-deleted pancreatic cancer cells was increased compared to wild-type CK19 mRNA expression (Figure 1(c)). This suggests that autophagy deletion strengthened the duct phenotype of pancreatic cancer. Next, we evaluated whether autophagy deletion influences pancreatic cancer cell viability using alamarBlue. Autophagy deletion increased cell viability during starvation, which is a common stimulus that induces autophagy (Figure 1(d)). Next, we investigated the relationship between autophagy deletion and the mitochondria. Autophagy suppresses cellular metabolism by degrading uncoupled mitochondria during mitophagy. Administration of the mitochondria uncoupler CCCP increased cell viability (Figure 1(e)). alamarBlue staining, which reveals mitochondrial metabolism, suggested a deficiency in the degradation of mitochondria. To evaluate morphological changes in the mitochondria, we performed immunostaining of TOM20, which is present inside the mitochondrial inner membrane. MIA PaCa-2 cell mitochondria were localized in the perinuclear region and fused (Figure 1(g)). CCCP administration resulted in fission of the mitochondria in wild-type pancreatic cancer cells and their subsequent diffusion throughout the cytoplasm. However, in autophagy deletion pancreatic cancer cells, mitochondria remained localized in the perinuclear region and were generally fused (Figure 1(g)). This suggests that autophagy in pancreatic cancer cells is necessary for morphological changes in uncoupled mitochondria. Next, to investigate the differences in responsiveness to anticancer agents following autophagy deletion, a common anti-pancreatic cancer drug, gemcitabine, was administered. Cell viability increased in autophagy-deleted pancreatic cancer cells (Figure 1(f)). Additionally, wild-type mitochondria exhibited fission and diffusion throughout the cytoplasm. However, we observed no morphological changes in the mitochondria due to gemcitabine in autophagy-deleted cells (Figure 1(g)). Pancreatic cancer showed increased cell viability after gemcitabine treatment because of autophagy deletion and the altered mitochondria morphology. This suggests that the response of pancreatic cancer to gemcitabine involves mitochondrial uncoupling and that mitophagy occurs during this process. It also suggests that the response of pancreatic cancer to gemcitabine requires the involvement of mitophagy.

### 3.2. Autophagy Deletion Acinar Cells

**Figure 2:**
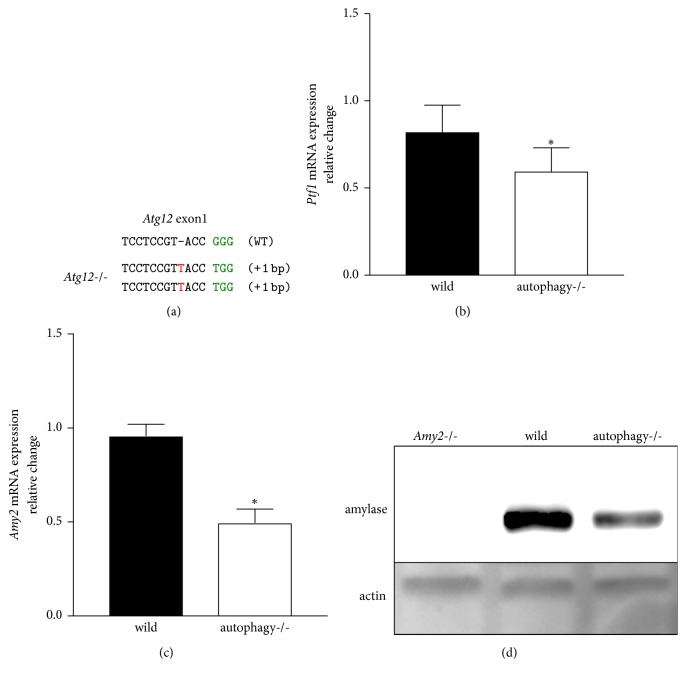
Autophagy deletion acinar cells. (a)* Atg12*-deficient AR42J genomic DNA sequence. Bases shown in red are indels (insertions and deletions). PAM sequence is shown in green. (b)* Ptf1* mRNA quantitative reverse transcription PCR. The vertical axis is the fold change relative to the GAPDH control. (c) Amylase mRNA quantitative reverse transcription PCR. (d) Western blotting results. Amylase(−/−) was used as a negative control. *β*-Actin was the loading control ^*∗*^*P* < 0.05 represents statistical significance.

Next, we used AR42J cells to investigate the relationship between the acinar phenotype and autophagy. AR42J is the only cell line that produces pancreatic enzyme proteins and possesses an acinar cell phenotype. We knocked out* Atg12* in AR42J cells using CRISPR/Cas9 (Figure 2(a)). Western blotting and immunostaining of LC3 were performed to demonstrate that* Atg12*-deficient AR42J was deficient in autophagy (Supp. Figures 2A, and 2B). Accumulation of LC3-II due to starvation, a general autophagy-induced stimulus, was decreased in* Atg12*-deficient cells. During immunostaining, LC3 also remained diffuse, and LC3 puncta decreased due to starvation. Autophagy deletion decreased the mRNA expression of* Ptf1*, an acinar cell marker [29], compared to the expression in wild-type cells (Figure 2(b)). Loss of Ptf1 causes the loss of acinar cells* in vivo* [30]. We evaluated amylase, a pancreatic enzyme and acinar cell phenotype marker, in the same manner. Amylase mRNA expression was decreased in autophagy deletion cells compared to that in wild-type cells (Figure 2(c)). Western blotting revealed decreased amylase protein levels (Figure 2(d)), suggesting that autophagy deletion weakens the acinar cell phenotype. The results in AR42J and MIA PaCa-2 cells suggest that autophagy deletion is necessary to strengthen the duct phenotype in pancreatic cancer cells and decrease the acinar phenotype in acinar cells.

### 3.3. Amylase Deletion Acinar Cells

**Figure 3:**
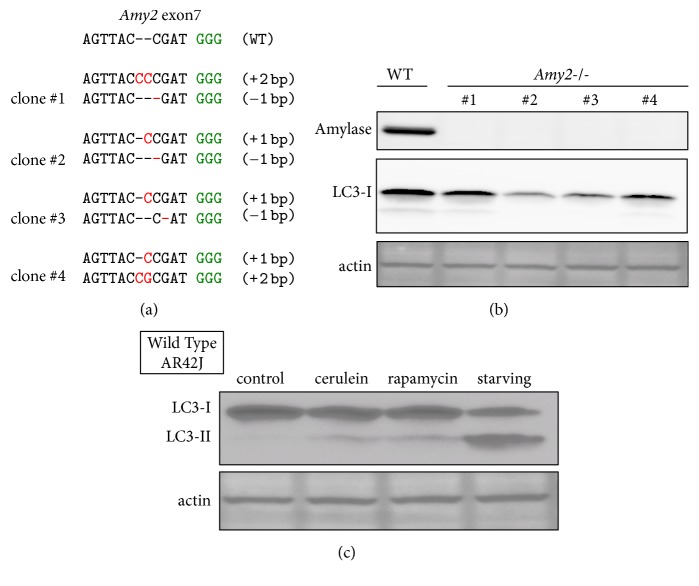
Amylase deletion acinar cells. (a) Genomic DNA sequence of independent 4 clones in which* Amy2* was deleted. Bases shown in red are indels (insertions and deletions). PAM sequence is shown in green. (b) Amylase-deficient AR42J western blotting results for 4 independent amylase-deficient AR42J clones. LC3-I is a precursor protein to the autophagy marker LC3-II. *β*-Actin was the loading control. (c) Wild-type AR42J was incubated with standard medium, 100 nM caerulein, or 100 nM rapamycin for 3 h, or in starvation medium for 1 h. LC3-II is an autophagy marker.

Next, to investigate the functional role of amylase, we knocked out amylase (Figures 3(a) and 3(b)). Amylase is an acinar cell marker; however, its functional role in cells is unknown. We established 4 independent amylase deletion clones. In normal culture, amylase deficiency did not lead to an increase in LC3-II, an autophagy marker (Figure 3(b)). LC3-I levels varied before being converted to LC3-II, but LC3-I is not an autophagy marker. On the other hand, amylase-deficient cells exhibited autophagy induction equivalent to that in wild-type cells during starvation (data not shown). Next, we observed an increase in LC3-II, an autophagy marker, in wild-type AR42J under starvation conditions as well as following treatment with rapamycin, a common autophagy-inducing agent (Figure 3(c)). Cerulein is the most common drug used to experimentally induce pancreatitis and induced autophagy in wild-type AR42J cells (Figure 3(c)). Cerulein-induced autophagy in wild-type AR42J is consistent with observations in a previous study [31].

### 3.4. Inducing Autophagy in Amylase Deletion Acinar Cells

**Figure 4:**
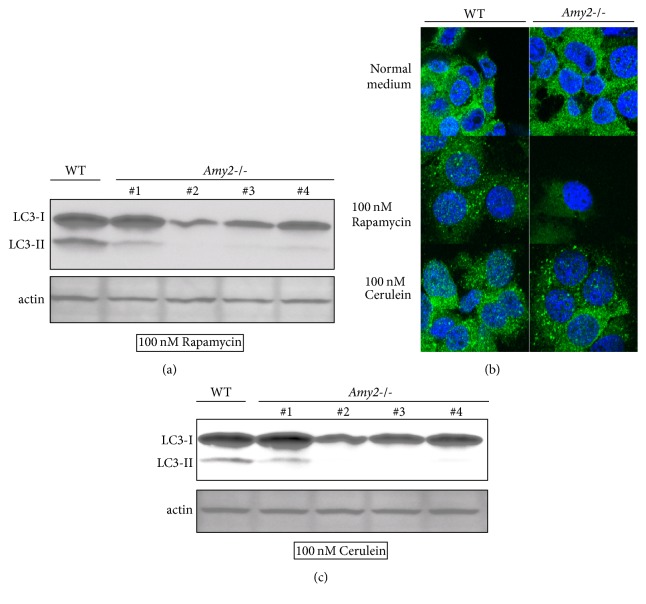
Amylase deletion and autophagy. (a) Wild-type and* Amy2*-deficient clones were incubated in 100 nM rapamycin for 3 hr. Western blotting results. LC3-II is an autophagy marker. (b) Wild-type and* Amy2*-deficient AR42J cells were assayed by immunofluorescence and observed by confocal microscopy. Green is LC3; blue is Hoechst stain (c) Wild-type and* AMY2*-deficient clones were incubated in 100 nM cerulein for 3 h. Western blotting results.

Next, we investigated whether amylase deletion influences the increase in autophagy caused by rapamycin and cerulein. We investigated the effect of rapamycin, a common autophagy-inducing agent. Compared to the wild-type, the* Amy2*-deficient clone showed decreased levels of LC3-II (Figure 4(a)). Next, we evaluated autophagy by morphological analyses. We examined LC3 by confocal microscopy. We evaluated autophagy by assessing LC3 puncta. Compared to the wild-type, the amylase deletion clone showed a decrease in LC3 puncta (Figure 4(b), Supp.  Figure 3C). These findings indicate that amylase increases rapamycin-induced autophagy. Next, we investigated the effect of cerulein, a pancreatitis-inducing agent. We observed less LC3-II in* Amy2*-deficient clones than in wild-type clones (Figure 4(c)). Moreover, in* Amy2*-deficient clones, we did not detect an increase in LC3 puncta (Figure 4(b), Supp.  Figure 3C). These findings indicate that amylase increases cerulein-induced autophagy.

Finally, we evaluated whether attenuation of autophagy by* Amy2* deficiency affects cell function. Cell viability was measured using alamarBlue (Supp. Figures 3A, and 3B). Cell viability, following rapamycin and cerulean exposure, was lower with respect to amylase deficiency than that in wild-type cells. Based on these findings, amylase deficiency attenuates cerulein-induced autophagy and decreases cell viability.

## 4. Conclusions

In this study, we found that autophagy deletion in a pancreatic cancer cell line increased the duct phenotype in pancreatic cancer cells and decreased the acinar phenotype in acinar cells; morphological changes in the mitochondria were also observed. This is consistent with previous studies of autophagy deletion using a GEMM. To further elucidate the influence of autophagy deletion on pancreatic cancer cells in vivo, xenograft experiments and assessments of mitochondrial metabolism need to be performed. Cell line knockdown experiments showed that autophagy-inhibiting agents are useful for pancreatic cancer treatment, and clinical trials are being conducted. However, in studies using GEMM knockouts, autophagy-inhibiting agents increased the rate of tumor-related death. Our results using cell line autophagy knockouts support the efficacy of therapeutic drugs targeting pancreatic cancer mitophagy.

A limitation of this study is that because the CRISPR/Cas9 system was used, off-target effects may have occurred. Although we created 4 clones of amylase-deficient cells, only a single* ATG12*-deficient cell clone could be produced. At least 2 clones are desirable for confirmation purposes. Additionally, expression of CK19 was not observed in* Atg12*-deficient AR42J cells. This was because of a decrease in the acinar phenotype marker, but we could not confirm that a shift to the duct phenotype had occurred.

We showed that amylase is necessary for the induction of autophagy by pancreatitis in acinar cells. Autophagy of organelles such as the mitochondria and the endoplasmic reticulum occurs in a selective manner, known as organellophagy. In the most well-known organellophagy, mitophagy, the mitochondrial protein PINK is important for selectivity [32]. In pancreatitis, selective autophagy occurs in pancreatic enzyme vesicles [31]. However, the specific proteins among those present in the enzyme vesicles, involved in pancreatitis-induced autophagy are not clearly known. As amylase is the most abundant protein in pancreatic enzyme vesicles, our results suggest the possibility that amylase selectively contributes to the pancreatitis-induced autophagy of pancreatic enzyme vesicles.

Amylase expression is eliminated during pancreatic cancer development. We found that autophagy in acinar cells is controlled by amylase, suggesting that loss of amylase expression contributes to pancreatic cancer development through autophagy.

## Abbreviations

CRISPR: Clustered regularly interspaced short palindromic repeats
Cas9: CRISPR-associated protein 9
LC3: Microtubule-associated protein-1 light chain 3
LAMP: Lysosome-associated membrane protein.

## Supplementary Materials

Supplementary 1 Supplementary Figure 1: (A) Western blotting of normal state wild-type and* ATG12*-/- MIA PaCa-2 cell lysate. ATG12 formed a complex with ATG5. (B) Result of immunostaining. Green is LC3; blue is Hoechst.

Supplementary 2 Supplementary Figure 2: (A) Western blotting of lysate from wild-type and* Atg12*-/- AR42J. LC3-II is a marker of autophagy. (B) Results of immunostaining. Green is LC3; blue is Hoechst. (C) Results of cell viability measurement.

Supplementary 3 Supplementary Figure 3: (A, B) Results of cell viability measurement. (C) Table displaying the results of immunostaining. The total counted cells, total LC3 puncta counted, and % of cells with positive LC3 puncta are displayed.

## References

[1] Howes N., Neoptolemos J. P. (2002). Risk of pancreatic ductal adenocarcinoma in chronic pancreatitis. *Gut*.

[2] Krejs G. J. (2010). Pancreatic cancer: epidemiology and risk factors. *Digestive Diseases*.

[3] Storz P. (2017). Acinar cell plasticity and development of pancreatic ductal adenocarcinoma. *Nature Reviews Gastroenterology & Hepatology*.

[4] Kopp J. L., von Figura G., Mayes E. (2012). Identification of Sox9-dependent acinar-to-ductal reprogramming as the principal mechanism for initiation of pancreatic ductal adenocarcinoma. *Cancer Cell*.

[5] De Lisle R. C., Logsdon C. D. (1990). Pancreatic acinar cells in culture: Expression of acinar and ductal antigens in a growth-related manner. *European Journal of Cell Biology*.

[6] Means A. L., Meszoely I. M., Suzuki K. (2005). Pancreatic epithelial plasticity mediated by acinar cell transdifferentiation and generation of nestin-positive intermediates. *Development*.

[7] Gruber R., Panayiotou R., Nye E., Spencer-Dene B., Stamp G., Behrens A. (2016). YAP1 and TAZ Control Pancreatic Cancer Initiation in Mice by Direct Up-regulation of JAK–STAT3 Signaling. *Gastroenterology*.

[8] Morvaridi S., Dhall D., Greene M. I., Pandol S. J., Wang Q. (2015). Role of YAP and TAZ in pancreatic ductal adenocarcinoma and in stellate cells associated with cancer and chronic pancreatitis. *Scientific Reports*.

[9] Chiari H. (1896). Uberdie Selbstverdauung des menschlichen Pankreas. *Zeitschriftfür Heilkunde*.

[10] Hofbauer B., Saluja A. K., Lerch M. M. (1998). Intra-acinar cell activation of trypsinogen during caerulein-induced pancreatitis in rats. *American Journal of Physiology-Gastrointestinal and Liver Physiology*.

[11] Dawra R., Sah R. P., Dudeja V. (2011). Intra-acinar trypsinogen activation mediates early stages of pancreatic injury but not inflammation in mice with acute pancreatitis. *Gastroenterology*.

[12] Mareninova O. A., Hermann K., French S. W. (2009). Impaired autophagic flux mediates acinar cell vacuole formation and trypsinogen activation in rodent models of acute pancreatitis. *The Journal of Clinical Investigation*.

[13] Hashimoto D., Ohmuraya M., Hirota M. (2008). Involvement of autophagy in trypsinogen activation within the pancreatic acinar cells. *The Journal of Cell Biology*.

[14] Mareninova O. A., Sendler M., Malla S. R. (2015). Lysosome-Associated Membrane Proteins (LAMP) Maintain Pancreatic Acinar Cell Homeostasis: LAMP-2-Deficient Mice Develop Pancreatitis. *Cellular and Molecular Gastroenterology and Hepatology*.

[15] Antonucci L., Fagman J. B., Kim J. Y. (2015). Basal autophagy maintains pancreatic acinar cell homeostasis and protein synthesis and prevents ER stress. *Proceedings of the National Acadamy of Sciences of the United States of America*.

[16] Rosenfeldt M. T., O'Prey J., Morton J. P. (2013). P53 status determines the role of autophagy in pancreatic tumour development. *Nature*.

[17] Tsukada M., Ohsumi Y. (1993). Isolation and characterization of autophagy-defective mutants of Saccharomyces cerevisiae. *FEBS Letters*.

[18] Fujii S., Mitsunaga S., Yamazaki M. (2008). Autophagy is activated in pancreatic cancer cells and correlates with poor patient outcome. *Cancer Science*.

[19] Yang S. H., Wang X., Contino G. (2011). Pancreatic cancers require autophagy for tumor growth. *Genes & Development*.

[20] Chude C. I., Amaravadi R. K. (2017). Targeting autophagy in cancer: Update on clinical trials and novel inhibitors. *International Journal of Molecular Sciences*.

[21] Cong L., Ran F. A., Cox D. (2013). Multiplex genome engineering using CRISPR/Cas systems. *Science*.

[22] Mali P., Yang L., Esvelt K. M. (2013). RNA-guided human genome engineering via Cas9. *Science*.

[23] Whitcomb D. C., Lowe M. E. (2007). Human pancreatic digestive enzymes.. *Digestive Diseases and Sciences*.

[24] Yan S., Wu G. (2016). Analysis on evolutionary relationship of amylases from archaea, bacteria and eukaryote. *World Journal of Microbiology and Biotechnology*.

[25] Buisson G., Duée E., Haser R., Payan F. (1987). Three dimensional structure of porcine pancreatic alpha-amylase at 2.9 A resolution. Role of calcium in structure and activity.. *EMBO Journal*.

[26] Ran F. A., Hsu P. D., Wright J., Agarwala V., Scott D. A., Zhang F. (2013). Genome engineering using the CRISPR-Cas9 system. *Nature Protocols*.

[27] Mizushima N., Noda T., Yoshimori T. (1998). A protein conjugation system essential for autophagy. *Nature*.

[28] Hanada T., Noda N. N., Satomi Y. (2007). The Atg12-Atg5 conjugate has a novel E3-like activity for protein lipidation in autophagy. *The Journal of Biological Chemistry*.

[29] Krapp A., Knöfler M., Frutiger S., Hughes G. J., Hagenbüchle O., Wellauer P. K. (1996). The p48 DNA-binding subunit of transcription factor PTF1 is a new exocrine pancreas-specific basic helix-loop-helix protein. *EMBO Journal*.

[30] Krapp A., Knöfler M., Ledermann B. (1998). The bHLH protein PTF1-p48 is essential for the formation of the exocrine and the correct spatial organization of the endocrine pancreas. *Genes & Development*.

[31] Grasso D., Ropolo A., Lo Ré A. (2011). Zymophagy, a novel selective autophagy pathway mediated by VMP1-USP9x-p62, prevents pancreatic cell death. *The Journal of Biological Chemistry*.

[32] Narendra D., Tanaka A., Suen D. F., Youle R. J. (2008). Parkin is recruited selectively to impaired mitochondria and promotes their autophagy. *The Journal of Cell Biology*.

