# Supplementary figures and images for: Using CRISPR/Cas9 to Knock out Amylase in Acinar Cells Decreases Pancreatitis-Induced Autophagy
Source: Biomed Res Int. 2018 May 17;2018:8719397. doi: 10.1155/2018/8719397 (PMC5985122; doi:10.1155/2018/8719397)

## Slide 1
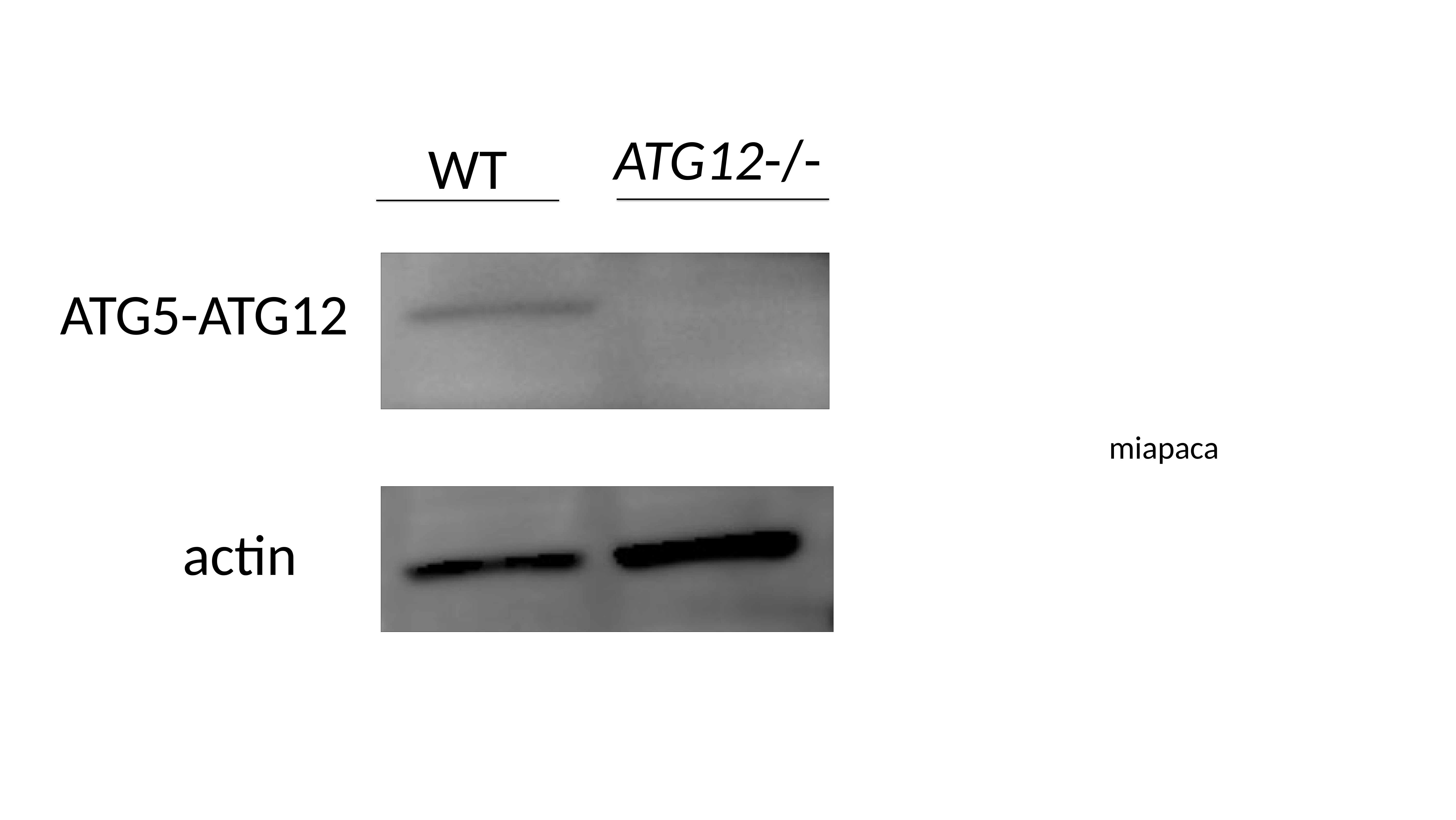

ATG12-/-
WT
ATG5-ATG12
actin
miapaca

Supplement: Supplementary 1 — Supplementary Figure 1: (A) Western blotting of normal state wild-type and ATG12-/- MIA PaCa-2 cell lysate. ATG12 formed a complex with ATG5. (B) Result of immunostaining. Green is LC3; blue is Hoechst. [file 8719397.f1.zip › suppl 1a_BMRI_2253658.pptx]

## Slide 1
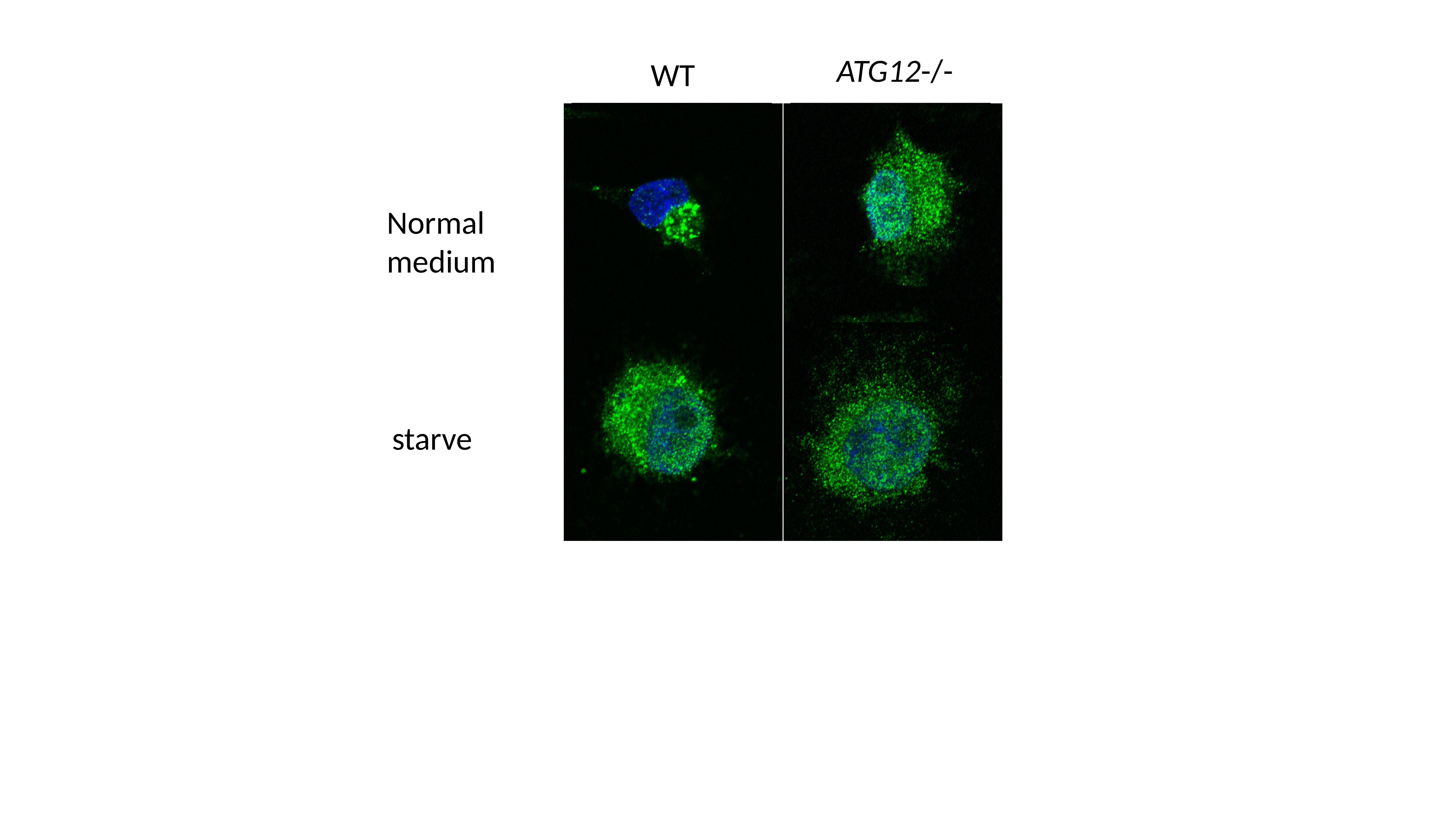

ATG12-/-
WT
Normal
medium
starve

Supplement: Supplementary 1 — Supplementary Figure 1: (A) Western blotting of normal state wild-type and ATG12-/- MIA PaCa-2 cell lysate. ATG12 formed a complex with ATG5. (B) Result of immunostaining. Green is LC3; blue is Hoechst. [file 8719397.f1.zip › suppl 1b_BMRI_2253659.pptx]

1hr starve  
cell viability

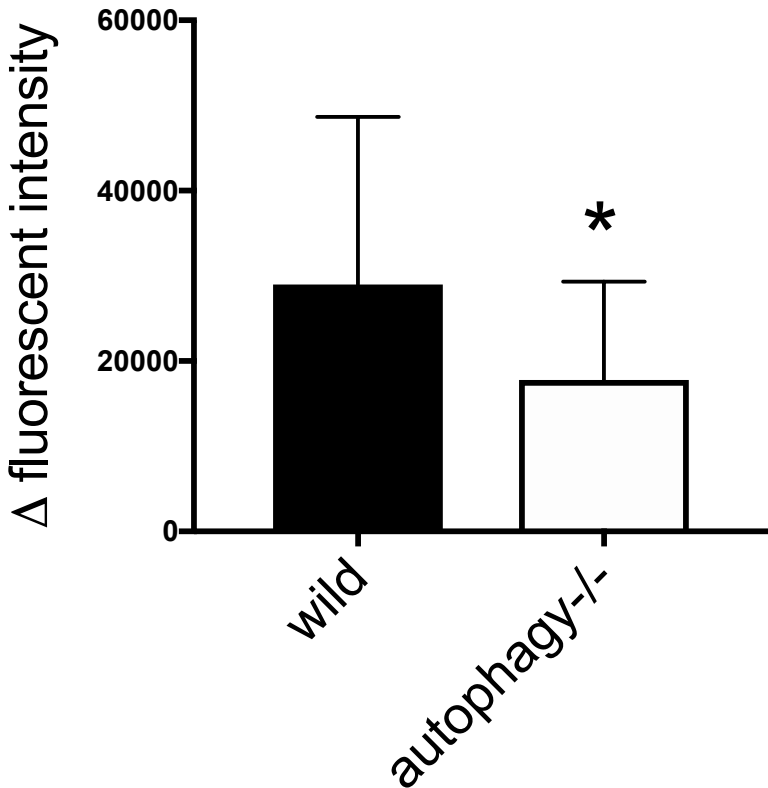

Supplement: Supplementary 2 — Supplementary Figure 2: (A) Western blotting of lysate from wild-type and Atg12-/- AR42J. LC3-II is a marker of autophagy. (B) Results of immunostaining. Green is LC3; blue is Hoechst. (C) Results of cell viability measurement. [file 8719397.f2.zip › supp 2C left_BMRI_2253663.pdf]

# 1hr starve cell viability

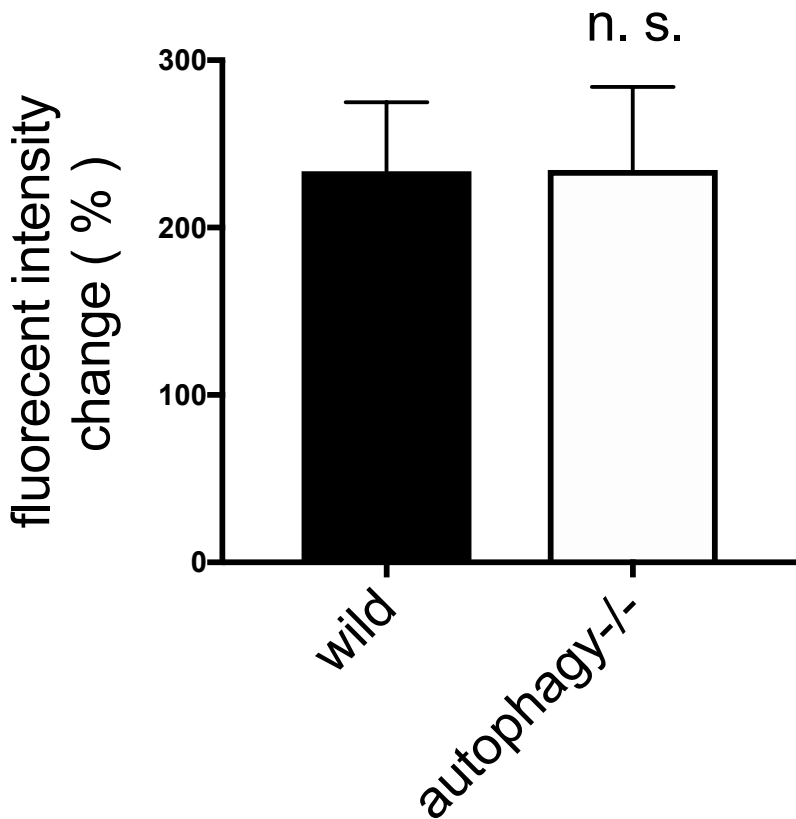

Supplement: Supplementary 2 — Supplementary Figure 2: (A) Western blotting of lysate from wild-type and Atg12-/- AR42J. LC3-II is a marker of autophagy. (B) Results of immunostaining. Green is LC3; blue is Hoechst. (C) Results of cell viability measurement. [file 8719397.f2.zip › supp 2C right_BMRI_2253666.pdf]

## Slide 1
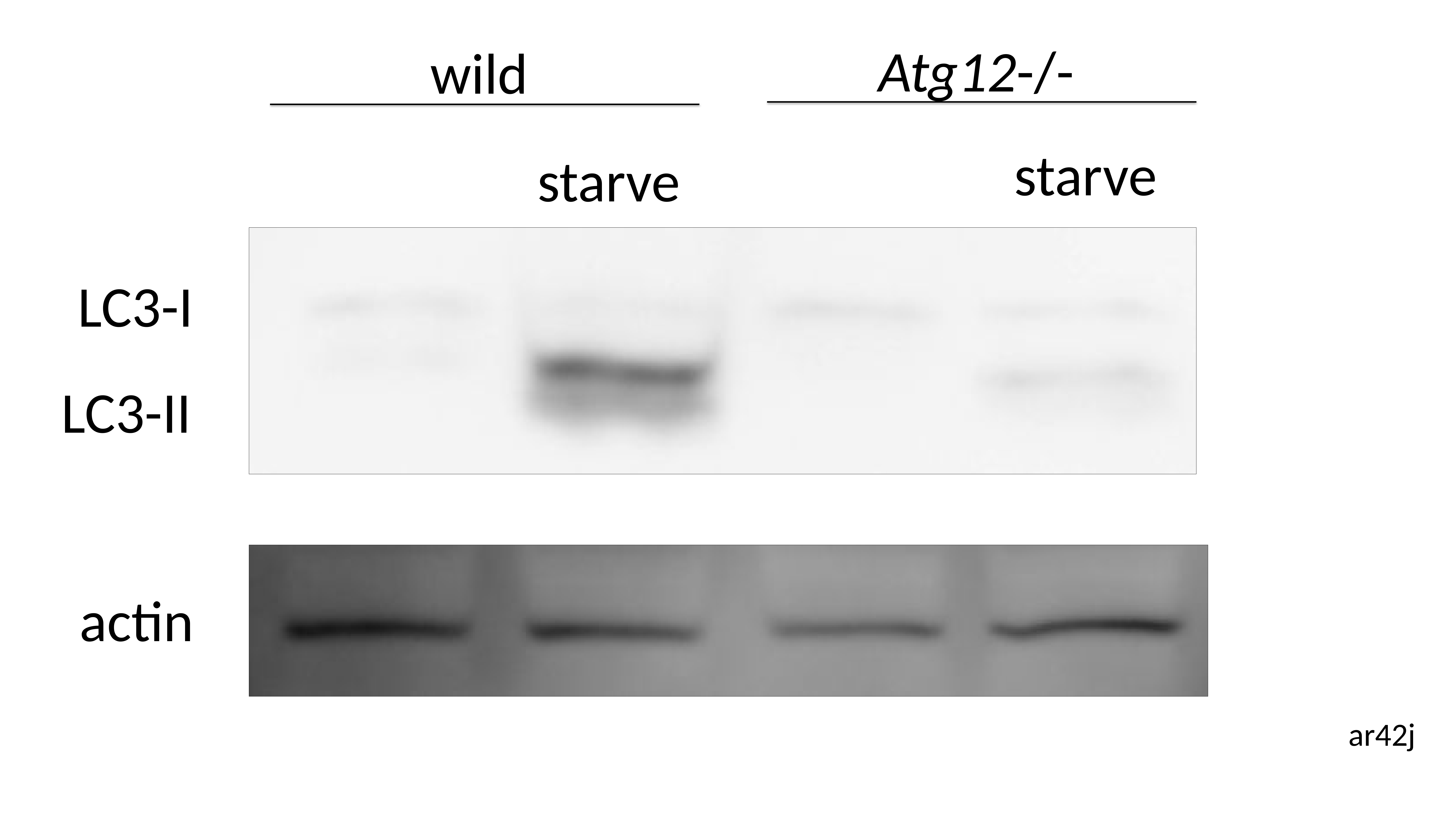

Atg12-/-
wild
starve
starve
LC3-I
LC3-II
actin
ar42j

Supplement: Supplementary 2 — Supplementary Figure 2: (A) Western blotting of lysate from wild-type and Atg12-/- AR42J. LC3-II is a marker of autophagy. (B) Results of immunostaining. Green is LC3; blue is Hoechst. (C) Results of cell viability measurement. [file 8719397.f2.zip › suppl 2a_BMRI_2253660.pptx]

## Slide 1
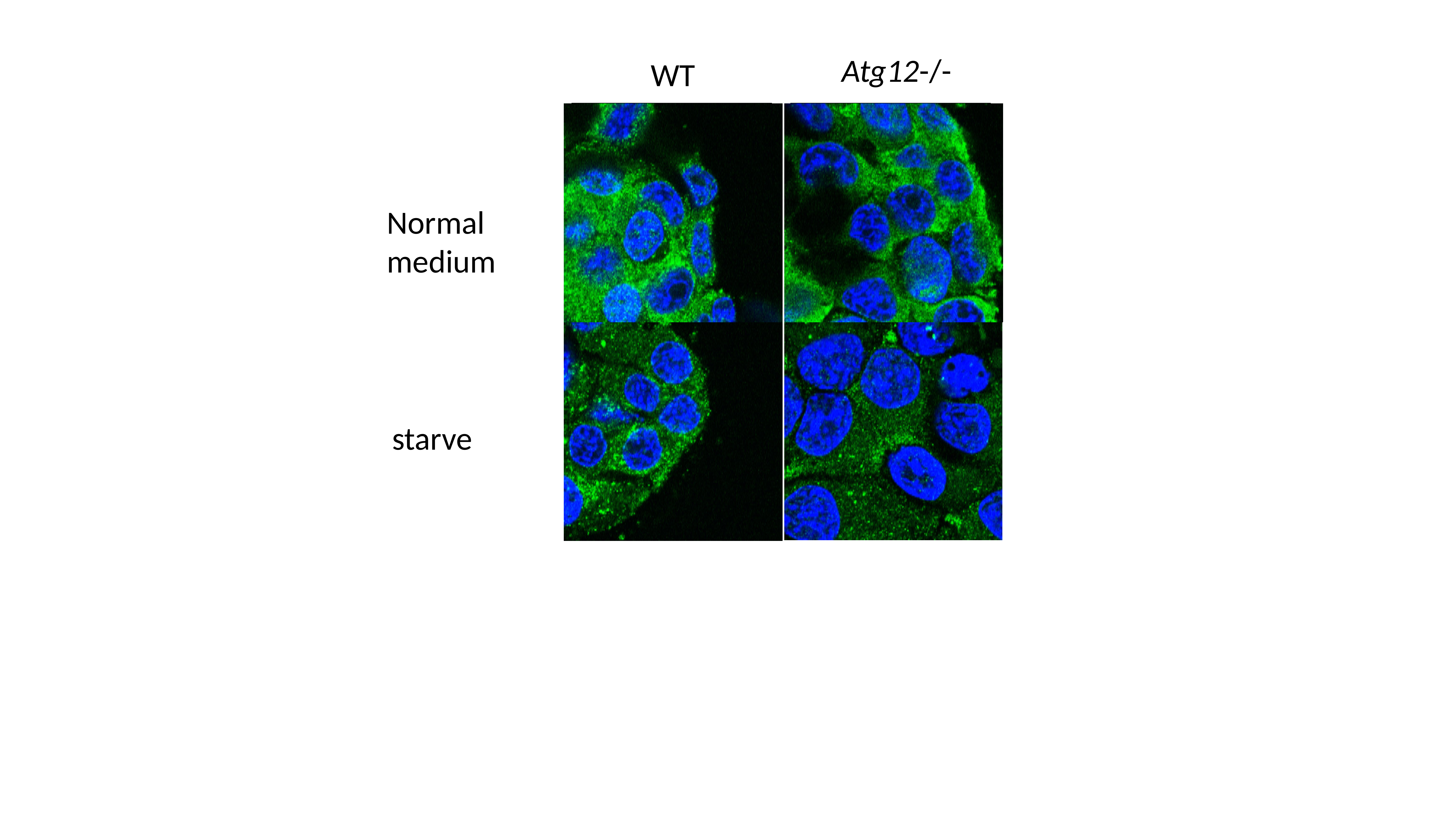

Atg12-/-
WT
Normal
medium
starve

Supplement: Supplementary 2 — Supplementary Figure 2: (A) Western blotting of lysate from wild-type and Atg12-/- AR42J. LC3-II is a marker of autophagy. (B) Results of immunostaining. Green is LC3; blue is Hoechst. (C) Results of cell viability measurement. [file 8719397.f2.zip › suppl 2b_BMRI_2253661.pptx]

# 100nM Rapamycin cell viability

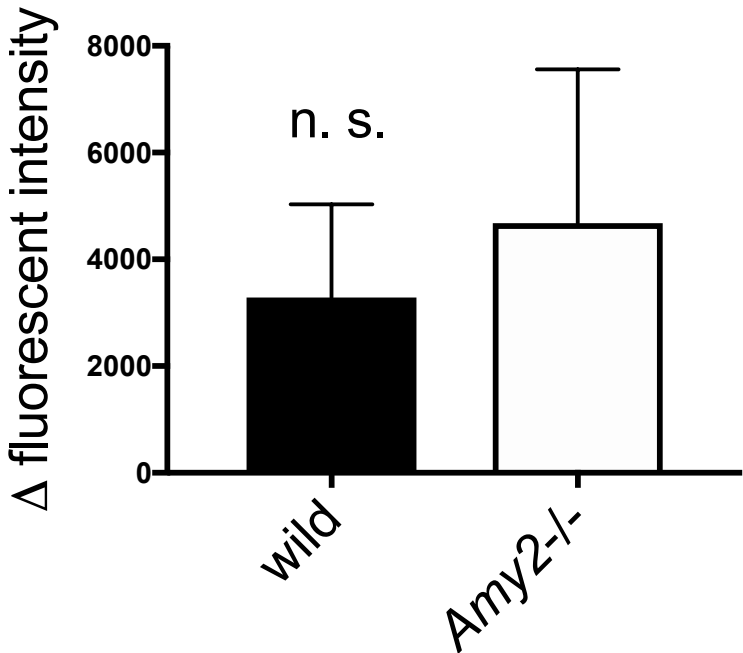

Supplement: Supplementary 3 — Supplementary Figure 3: (A, B) Results of cell viability measurement. (C) Table displaying the results of immunostaining. The total counted cells, total LC3 puncta counted, and % of cells with positive LC3 puncta are displayed. [file 8719397.f3.zip › supp 3A left_BMRI_2253669.pdf]

# 100nM Rapamycin cell viability

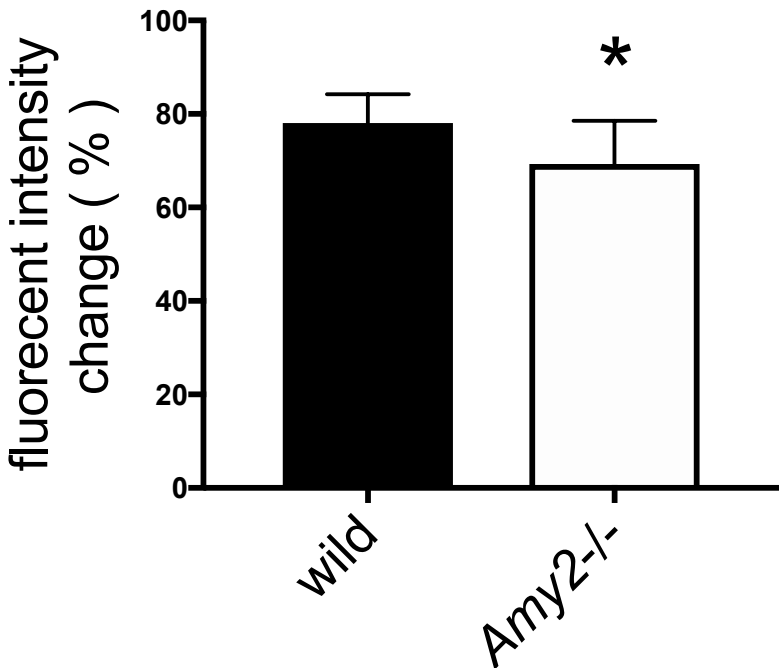

Supplement: Supplementary 3 — Supplementary Figure 3: (A, B) Results of cell viability measurement. (C) Table displaying the results of immunostaining. The total counted cells, total LC3 puncta counted, and % of cells with positive LC3 puncta are displayed. [file 8719397.f3.zip › supp 3A right_BMRI_2253674.pdf]

# 100nM Cerulein cell viability

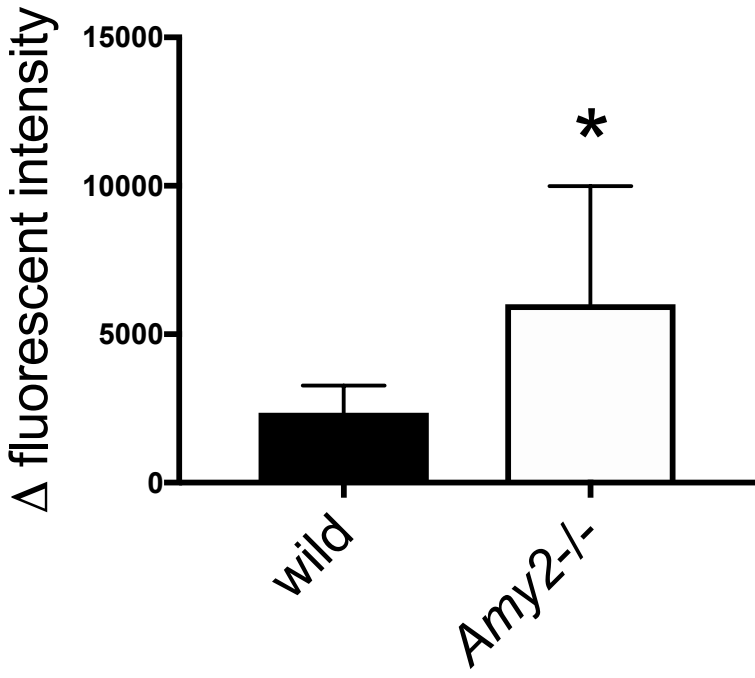

Supplement: Supplementary 3 — Supplementary Figure 3: (A, B) Results of cell viability measurement. (C) Table displaying the results of immunostaining. The total counted cells, total LC3 puncta counted, and % of cells with positive LC3 puncta are displayed. [file 8719397.f3.zip › supp 3b left_BMRI_2253677.pdf]

# 100nM Cerulein cell viability

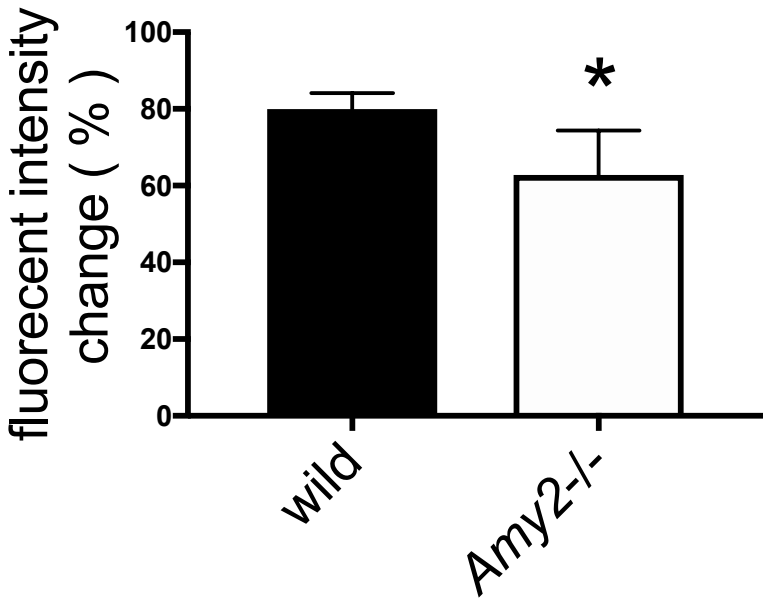

Supplement: Supplementary 3 — Supplementary Figure 3: (A, B) Results of cell viability measurement. (C) Table displaying the results of immunostaining. The total counted cells, total LC3 puncta counted, and % of cells with positive LC3 puncta are displayed. [file 8719397.f3.zip › supp 3b right_BMRI_2253680.pdf]
