# Supplementary material for: Using CRISPR/Cas9 to Knock out Amylase in Acinar Cells Decreases Pancreatitis-Induced Autophagy
Source: Biomed Res Int. 2018 May 17;2018:8719397. doi: 10.1155/2018/8719397 (PMC5985122; doi:10.1155/2018/8719397)
Supplement: Supplementary 3 — Supplementary Figure 3: (A, B) Results of cell viability measurement. (C) Table displaying the results of immunostaining. The total counted cells, total LC3 puncta counted, and % of cells with positive LC3 puncta are displayed. [file 8719397.f3.zip › suppl 3C_BMRI_2253682.docx]

|  |  | Total cells | Total LC3 puncta | LC3 puncta positive cells(%) |
| --- | --- | --- | --- | --- |
| 100nM  Rapamycin | Wild | 30 | 5 | 16.6 |
|  | *Amy2*-/- | 58 | 6 | 10.3 |
| 100nM  Cerulein | Wild | 51 | 17 | 25.5 |
|  | *Amy2*-/- | 128 | 28 | 18.0 |
